# A View of the Formation, Discipline, and Economy of Armies

**Published:** 1845-10

**Authors:** 


### A VIEW OF THE FORMATION, DISCIPLINE AND ECONOMY OF ARMIES. By the late *Robert Jackson*, M.D. Inspector General of Army Hospitals. Third Edition, revised; with a Memoir of his Life and Services, drawn up from his own Papers and the Communications of his Survivors. 8vo. pp. 425. Parker and Co. Whitehall, 1845.

THE Editors—whoever they may be—of this new and greatly improved Edition of a most valuable work, have done good service to the public, and especially to the military public, by resuscitating from comparative oblivion a treatise which has been truly said to unfold, at every page, the feelings and impressions of the true soldier, together with the observation of the profound MILITARY PHILOSOPHER.

The author of this volume served in three British wars—was intimately acquainted with military history—and enjoyed opportunities of examining the condition of most of the great armies of Europe. He has recorded, as no other one has recorded, the character of the British soldier, moral and physical, in social life—in quarters—and in the shock of battle—and all this in language at once forcible, elegant, and dignified. In the language of Dr. Barnes, "this is a truly learned and excellent work. To princes and statesmen—to commanders and military officers of every rank, and of every country, this is a most instructive and useful book." He might have added that, to the military surgeon also, it is invaluable. Owing to several circumstances the work in question never had an extensive circulation. In the first place, although the author was a good classical scholar, and was acquainted with several modern languages, yet his style was not very inviting, and the book was printed in a clumsy quarto at a provincial press, without any of the ornaments that now embellish the literature of our times. The public too—whether military or naval, was not such a reading public as at the present day. From these and other causes, the Essay on Armies was comparatively little known, and the present edition, enriched as it is by Notes from the author's own pen, will have a wide circulation and exercise a dominant influence.

The biography, too, which extends to 120 pages, is original, or nearly so, and, as much of it is taken from posthumous notes, it is in a considerable degree *autobiographical*. The editors, however, have drawn valuable materials from various sources—much from Dr. Borland—to enrich and illustrate the life of their favourite author—the whole being worked into an eloquent composition of which a PLUTARCH might be proud.

It is justly observed by the editors that those who rise to fame and fortune in the medical profession must be possessed of intrinsic talents and unwearied industry. Its members are drawn from the middle classes of society, and have neither patronage from the nobility, nor lucrative situations to look up to in their thorny journey through a laborious life. These truths are well exemplified in the history of our present author.

ROBERT JACKSON was the son of a small farmer in Lanarkshire, and born (1750) near the falls of the Clyde. His father, however, gave him a good preliminary education, and in due time apprenticed him to Mr. Baillie, a country surgeon of some distinction. In three years he repaired to Edinburgh (1768) to pursue his professional studies under the Monros, Cullens, Blacks, &c. But the *res angusta domi* did not enable the young aspirant to pursue his studies continuously here, and he was forced to take a cruise in a whaler every summer, to supply the scanty means of paying his way in the winter classes! Our author was thus early compelled to combine the practice with the theory of his profession—a circumstance that probably influenced, in no inconsiderable degree, his habits of observation in future life.

After three or four years' of winter study and Greenland practice, he determined to try the West Indies, and debarking in Jamaica he became assistant to Dr. King, of Savanna-la-mar, where he had the charge of a detachment of soldiers in the military barracks there, and first commenced his observation of a soldier's character, habits and diseases. But he was disgusted from the beginning with the contemplation of slavery, and determined to quit a land where it existed. After four years' residence, therefore, in Jamaica, he embarked for America, but being obliged to debark, for want of a certificate, he travelled on foot across the whole island, at the imminent risk of his life in such a horrid climate.

At length he found himself at New York, in the year 1788—with little more than a few shillings, a Homer, and a Greek Testament in his pockets! He tried to get a Commission in the New York Volunteers, but, in the mean time, he was reduced to almost starvation. In this forlorn condition, he offered himself as a volunteer in the 71st Regiment, when Col. Campbell, struck by his appearance, received him, first as a military volunteer, into the regiment, and placed him afterwards as an assistant to the surgeon (Dr. Stewart), who treated him with kindness.

"Furnished with the not very luxurious outfit of a soldier's tent, blanket, and ration, he never in his life, perhaps, enjoyed a day of more unalloyed and hearty content, as he was often heard to declare, than when he joined the 71st. Though reclining on a bundle of straw, and after a dinner of which salt pork had formed the whole bill of fare, he felt comparatively as if he had been translated to Paradise. Rescued as he was from indigence and starvation, he had now the proud and blessed conviction, that he was to earn his bread by the humble exertion of the talents God had bestowed upon him; for most devoutly through life did he aspirate the wish of one of his gifted countrymen, whose lines of life had not fallen in pleasant places:

'Thy spirit, Independence, let me share,
Lord of the lion-heart and eagle-eye;
Thy steps I follow, with my bosom bare,
Nor heed the storm that howls along the sky.'"

Although a Commission was offered him in the New York Volunteers afterwards, he determined to remain in the Seventy-first. The men becoming very sickly he was attached to the hospital department at Knightsbridge, where he proposed and effected most beneficial changes in the food of the sick which, at that time, was the same in kind (salt pork and salt beef) as of the healthy! In this situation he evinced great courage as well as great generosity.

"During the heat of the action fought at Cowpens by a division of the British army, under disadvantages of unfavourable position and numerical inferiority, at a moment when the issue of the battle was no longer doubtful; Mr. Jackson, who happened to be well-mounted, perceiving that the horse of the officer commanding the British troops had been shot under him, immediately rode up to the dismounted commander, and tendered to him the horse he was riding himself, remarking, that for his own part he was but an obscure individual, whose escape could have but little beneficial influence, but that *his* (the officer's) safety was of the highest importance to the army. The commander, Colonel (afterwards General) Tarleton, thus pressed, accepted, though reluctantly, the generous offer, and escaped." P. xxxiv.

It is not a little curious that Col. Tarleton, in his history of this portion of the war, has entirely forgotten—or at least passed over—this generous action of our author! Dr. Jackson himself has not recorded it in any of his works; but it has been attested by an eye-witness (Colonel Hovenden), and acknowledged as a fact by the author, when questioned on the subject.

Mr. Jackson, being made a prisoner by the Americans, was well treated, and employed himself in tending the sick of both armies. Washington was so much pleased with our author, that he released him from prison without requiring even his parole.

Returning to Scotland in 1782, he started for London on foot, like his countryman Smollett, and soon afterwards made a tour through a considerable portion of Europe, in the pedestrian fashion, but without the advantage which Goldsmith possessed—a German flute. Many of our author's adventures on this whimsical and romantic enterprize are curious and amusing; but we dare not delay the thread of our narrative. After a ramble of seven or eight months, our author landed at Southampton with four shillings in his pocket, after walking upwards of five thousand miles, on the most scanty finances. He staid but a short time in London, when he set out on foot, in the middle of winter, for Perth, where his original regiment, the 71st, was disbanding. He travelled through the Highlands to the Isle of Skye in his favourite pedestrian style, and, on returning to Edinburgh, he married a lady of fortune and accomplishments —the daughter of Dr. Stephenson, with whom he went to Paris to study, and afterwards graduated at Leyden—not by purchase, but by actual trial and examination. Returning to England, he settled in practice at Stockton-on-Tees, where he became a popular physician, but he never took heartily to private practice.

> "Our profession (says he) is a lottery, and requires something beyond knowledge to lead to success. I like it in an hospital; I do not like it as a country practitioner, and I do not find I can practise it with success. The cases generally are in advanced stages before I see them." P. lix.

There is some truth in this. The Physician is like the Poet—"nascitur non fit." He became an accomplished scholar and linguist, devoting all his spare time to classic and scientific study in the dead and living languages.

In 1791, he published he result of his observations on fever in the West Indies and America, which attracted great attention at the time. In 1793, when the war broke out with France, he volunteered to serve as a regimental surgeon in the West Indies. But the death of John Hunter, in the mean time, placed the army medical department under a Board, which procured an enactment, that army surgeons were incapable of holding the rank of army-physicians!! Dr. J. remonstrated with Sir Lucas Pepys, but to no purpose.

> "The physician-general lost temper, and replied with acrimony, 'Had you the knowledge of Sydenham or Radcliffe, you are the surgeon of a regiment, *and the surgeon of a regiment can never be allowed to be physician to his majesty's army.*' The words were pronounced with official authority, and a final emphasis, that showed there could be no appeal. The doctor contented himself with observing, that the regulation was made in ignorance, and could not fail of being injurious to the service in its consequences; a remark which a worldly-wise man perhaps would have left unsaid; but as it was the truth, he felt no scruple in stating it as his honest opinion; and thereby no doubt sealed his doom with the triarch of the Board." P. lxiv.

He accompanied the regiment to Flanders in 1794, and His Royal Highness the Duke of York became his patron, and ultimately promoted him, despite the Physician and Surgeon-General's regulations. His next appointment was to St. Domingo, where the duty assigned to him gave him ample means of investigating the causes and treatment of endemic diseases. Here he corrected many most enormous abuses, and saved his country hundreds of thousand in money, while he promoted the comforts of the sick.

Returning from the West Indies, he made a tour, in company with his friend Dr. Borland, through the United States, and there had fresh opportunities of pursuing his studies of fever. Arriving in England, he published the results of his experience, both in Europe and the West Indies. Having been appointed to the army-depôt-hospital at Chatham, he corrected numerous abuses; but the BOARD had a rod in pickle for him, and soon tried to pick a hole in his skirt, by stating, or, at all events, insinuating, that the mortality under his care was greater than it ought to be. Other insults were offered to our author, for which he could find no redress in any Court of Law; he, therefore, took the law into his own hand, and caned the Surgeon-general, Mr. Keate, in the street. For this assault he was tried, and sentenced to six months' imprisonment. This he bore with fortitude, not having defended the action, or even employed counsel to mitigate the damages. It is probable that, in these days, the penalty would not be more than a few pounds, when the provocation was properly delineated. This was in 1809, the year of the fatal Walcheren expedition.

"As mentioned by Dr. Jackson above, in 1810 the government instituted an important change in the constitution of the army medical department. The board, consisting of a physician-general, a surgeon-general, and an inspector-general, was dissolved. It was high time. It had either winked at, or fostered, a system of enormous abuse. The system of hospital management and discipline carried out by Dr. Jackson at the military depot of the Isle of Wight (now long since become that of the whole British army), was so simple, economical, and effective in all its parts, that it had struck dismay into the hearts of contractors and purveyors, who felt their occupation gone, under a rule of weekly audit, at which the wine and porter contractor, the medical stores and surgeons' instruments' contractor, the purveyor; in short, the entire host of harpies that had battened on the spoliation of the day, took the alarm. The very official existence of such a man as Jackson was ruin to them. This was only to be evaded by *his* ruin, and that they sought, after the fashion specified in the foregoing pages, to effect, with the aid of a not very scrupulous board, that passed enormous sums, say of seventy thousand pounds even, unaudited and unchecked! He stood the storm firm and undaunted, and strenuously demanded an investigation before a court martial, which the then commander-in-chief, Sir David Dundas, for reasons not known to us, declined to grant. But, as Napoleon would say, the destiny of the board was fulfilled—it ceased to reign. The management of the army medical department was now vested in a director-general of the whole, assisted by three principal inspectors, upon a plan analogous to that adopted in other branches of the military executive. P. lxxxix.

This opened the door for our enterprizing physician, who was appointed to the West India station, where he toiled again for years in the acquisition of knowledge respecting tropical diseases. The results of his observations are partly deposited in the Director-General's office, and partly published in his works. In 1815 he returned, and published his "*Sketch of the History and Cure of Febrile Diseases; more particularly as they appear in the West Indies, &c.*"—a work of great merit, and which alone would transmit his name to posterity. A second edition, enlarged, was printed in 1820. Although a rigid disciplinarian himself, Dr. Jackson disliked the rigid Prussian system of terror and degradation.

"Whether with the medical or military officer," he says, "the heart must be warm with charity, the mind firm in knowledge; for no class of men are more dexterous in probing the rotten parts of the heart, or in unmasking the weak mind of their superior, than the mass of common soldiers." He goes on to observe, "The physician restores the sick soldier to health; the military officer witnesses the process; he is in some degree the master of the means, and he is judge of the effect. The soldier who is consoled by the words of friendship, as he lies feeble and dejected in the hospital-bed, gives courage to the arm in the field when restored to the vigour of health, conquers like a hero, or falls by the side of his officer and friend—his wounds in front, and his face towards the enemy." P. xci.

Anxious to see what were called the yellow fevers of Spain and the Mediterranean, he travelled thither in search of them, but was disappointed by troubles and vexatious quarantines. He then sailed for Malta, and even Constantinople, in search of the Plague, which seems to have hidden its diminished head wherever he went. He returned by Greece, and indulged his classical associations and reminiscences at Athens and other celebrated places. But the yellow fever being his chief object of research, he came back to Cadiz, and on the very day of his arrival, the fever was declared—namely, on the 25th August. At Cadiz and Xeres he investigated the epidemic, and satisfied himself as to its nature and treatment. This he published next year, under the title of "Remarks on the Epidemic Yellow Fever of the South Coast of Spain."

"This the last though not the most toilsome of our philanthropist's adventures, evinces the prompt heroism which he was always ready to exhibit in the cause of science; for heroism it is in a man of three-score and ten to forego all considerations of domestic comfort, and to grapple with certain danger, and that in its least attractive form, in order to aid in the increase of knowledge, and the mitigation of human suffering. Science has its forlorn hopes, but they are not heard of in the annals of military glory, and not marked for reward in the tablets of the statesman. We seldom see such zeal and moral courage surpassed, even in the buoyancy and vigour of youth; but our admiration of both is enhanced, when we learn that in his seventy-seventh year, a few weeks before his death, he conveyed to the director-general of the army medical department an offer to waive his rank, and proceed to Portugal to do duty in the military hospitals under a junior, with the British force then acting in that country under the command of General Sir W. H. Clinton." P. civ.

By his first wife he had three sons and a daughter—all of whom, it is supposed, are dead. The youngest son died while his father was in St. Domingo, and it is not a little curious that the father dreamt of the death of his son on the very night of the fatal event. Although not superstitious, this catastrophe made a deep impression on the Doctor's mind.

But the longest life will have an *end*, and all our wanderings will terminate in the tomb. Few men have travelled more or observed better than our author. The line applied by the Roman Bard to Ulysses is strictly applicable to Dr. Jackson:—

"Et mores hominum multorum vidit et urbes."

Dr. Jackson died of a paralytic affection on the 6th of April, 1827, at Thursby, near Carlisle, in the 77th year of his age, attended by Dr. Barnes.

From an eloquent and animated sketch of our author's character by his talented biographers, we can only condense a few of the most prominent features for our readers.

Dr. Jackson's height was five feet eight inches—erect and muscular, with fair and florid complexion, blue eyes, expanded forehead, Grecian nose, and cheerful countenance. His dress was neat and plain, and his air was military. He was humane and liberal both in sentiment and action. Cruelty and harshness he detested—warm and sincere in his friendship, as was evinced by his promptly leaving his home and his practice at Stockton to attend upon his friend Dr. Borland, when ill of typhus fever in Kent. He was extremely temperate in diet, living chiefly on vegetables, and quenching his thirst with plain water, or *eau sucrée*. His veneration for pure Christianity rendered him indignant at anything approaching to a systematic corruption of its truths. He felt that devotion to the Supreme Being was the most powerful bond that keeps human nature in its right course. He never used tobacco in any form, and maintained that it was injurious to health.

"His views in regard to gestation, or exercise in the open air, and cold affusion in fever, were original and decided. Gestation in the open air, and change of air, were adopted systematically by him in the West Indies, North America, the continent of Europe, &c., in the last stages of protracted fever, and in all cases where fever proved intractable. This was done in camp or cantonment, as the case might be; and up to the present day no one has carried out this remedial measure with the same careful boldness, ability, and tact, as he did. His success with it astonished the army, and it is hoped will lead others to tread in his steps, though it may expose them to ridicule, as it did him. He used cold water affusion in the fevers of Jamaica largely between the years 1774 and 1778, and the proof that he did so is furnished by manuscript notes at the time transmitted to the late eminent Dr. Gregory, of Edinburgh. He preceded Dr. Wright in the use of this powerful curative agent by more than ten years, and Dr. Currie by more than twenty." P. cx.

Nurtured in the school of poverty, self-denial, and hardship, we find him steadily marching on, undismayed by the dark or rugged realities in prospective. Such a man has not lived in vain, had he only shewn the example in his own person, how the pilgrimage of the world is to be manfully gone through without sinking into the slough of despond.

"It has been observed to the honour of Bacon, that his scientific zeal in his old age led him, during the prevalence of severe snow, to expose himself to its inclemency, in order to analyze its nature, even at the risk of his health; what then shall be said of the venerable Jackson, who at threescore and ten, indifferent to household ease and comfort, goes on a far journey to investigate the laws of contagion, or who volunteers at seventy-seven to proceed on actual military service in the field, *under a junior?* P. cxi.

We have already noticed that Dr. Jackson's plans and suggestions saved the Government, in the colonial contract alone, more than eighty thousand pounds annually, while adding immensely to the health and comfort of the sick soldier. Surely his widow should not be neglected by the State in her declining years! Is she not better entitled to a pension than most of those who now enjoy such gifts from the country? We can only make room for one more extract.

"This is not the place to speak of his professional publications, which we briefly dismiss by saying, that they are characterized by vigour of thought, close observation of nature, and originality of views. The work, however, by which he will, if we mistake not, be best known to posterity, is the one before us. The reader who expects mere dry essays on military affairs, will be surprised as gratified in turning over its pages, to find that he is under the guidance of a masculine and profound intellect, which traces the progress of society throughout the world, inductively and admirably, while professing only to treat of the economy and discipline of armies. Already it has received the *imprimatur* of a hero's approbation—of him who was left 'alone in his glory' on the height of Corunna. The author has brought to his work the light of a wiser philosophy than was too often the fashion of his day. He demonstrates that the basis of all strength is religion, morality, and order, and that without these military power has no permanence of cohesion. He unfolds in a perspicuous manner grand views of national character, and the hosts of history are marshalled before us in more than review array. We have in his pages a clear insight into many motives, and a lucid estimate of the action and re-action of causes upon the human heart as wielded in mass for a power of offence or defence. One principle runs luminously through the whole, that *discipline* is the hoop that keeps together the staves of this great state vessel, military power; and that much indeed depends not merely upon the nature of the hoops, but the quality, seasoning, and fibre of the staves. An army should be carefully recruited, and if amenable to perfect discipline, and sensible to sympathies of patriotism and religious impression, it must be invincible." P. cxvi.

"We have thus endeavoured, as far as our materials permitted, to give a plain and intelligible account of a very uncommon man. He was rather with armies, than of them; that is, in regard to personal *status;* and yet, who has described what armies have been, are, and ought to be, so well? He was essentially a soldier in his feelings, habits, and objects, though a philosophic one, withal. He owed something to circumstances, but more to talent and integrity, and not a little to true genius. We never see him at a loss. In the barrack, the hospital, on the march, or in action with the enemy, he was ever useful, humane, efficient, and brave. He was always going out of self, and studying to benefit others to the utmost of his power. It was the same in private life. Friendship with him was a sacred and staunch bond—a living fountain, springing from the depths of his pure humanity. That he was not altogether overborne and crushed by a cruel system of persecution, was owing, in a great degree, to the salient energies of his own mind, and partly to the support of that excellent friend, whom his merits had bound to him with chords of steel, the right gallant and steadfast Sir Harry Calvert, and of the commander-in-chief, that high-minded, generous-hearted prince, who was, indeed, not only the soldier's friend, but the friend of the soldier's friend wherever he recognized him.

"The grave received Robert Jackson as a shock of corn in his season. He descended into it, not as is often the case, with long lingering ailments, tottering pace, faculties for years benumbed, affections blunted, and feelings withered. No, but with an eye to the last of unquenched vivacity and intelligence, a countenance glowing with benevolence, a mind still unsated and thirsting for science, and a heart as vividly alive to the claims of friendship and goodness, and as tenderly overflowing with pity for human suffering, as in the days of his prime. This was a man whom a king might have delighted to honor. Something, it may be conceived, in the way of distinction from the common herd, was due to him, as a man, and as an army medical-officer; were it only for his admirable system of hospital finance, which had saved hundreds of thousands to the state, and his sanatory measures that saved battalions from disease and death. Surely for one who had turned the power of subtle analysis so largely to the public good; who had said boldly in his place what it behoved a patriotic public servant to declare; surely a practical philosopher with such a far retrospectiveness taking a calm bird's-eye view of the historical horizon, or a just and penetrating one of the contemporary epoch, and giving his views to the world with such force, originality, freedom and freshness, merited some public mark of regard! Be that as it may, had he written nothing but the work before us, he would be acknowledged as a public benefactor; for who deserves the title better than he who, unfolding the volume of human nature, impresses the great lesson that what is merely aggressive and vicious, however strong for a time, will assuredly eventually perish; that only the defensive, the patriotic, and the right, will stand the shocks of time: and that nothing is truly strong, consistent, and enduring, but what is correct in principle, and scientific in its adjustments. He has written as with an iron pen upon the rock, so that all men, but especially his countrymen, may read and reflect upon the great truths each in his place, that without a never-sleeping discipline perfect in all its parts, and a sterling piety with its corslet of holiness, no country can defend itself with armies, and no armies can be eventually victorious. But promotion cometh not from the east or the west: it is often the result more of accident than a recognised principle. If it came from a fixed principle of doing honor to all to whom honor is due, without reference to aristocratic class-leanings and influence, then would it be unintelligible how a man like Jackson came to be so overlooked or wilfully neglected as he seems to have been. His name is not to be found in the distinguished, by Gazette. Why, we leave it to those who cherish this exclusive system to declare. If we ask, what order usually conceded to men of the people he would not have graced, we can easily anticipate the reply of the wise. If we ask, what the sovereign or the government did for such a truly noble and soldierly character, in the way of conferring distinction, the answer is as simple as was the cast of his own vigorous intellect, and amiable disposition—NOTHING! His only reward then was the internal one—a sufficient one for a good man perhaps, but for which he owes nothing to rank or power. The reflexion of this internal reward is all the honor his memory has as yet received, which will become enhanced as his works are better distributed, known, and appreciated. We find then that a man eminent for his public spirit, his services, his varied information and learning, brought up in a military school, and throwing out his ideas continually for military purposes of improvement; a man remarkable for his chivalric contempt of death in the cause of duty, no less than for his general disinterestedness, nice delicacy, and scrupulous integrity in every relation; a man esteemed highly too by many whose regard of itself stamped the honor and merit of the recipient; we find that seventeen years ago, one who was all this, died in England in comparative obscurity, unnoticed by the authorities of the day. All this happened, too, to one who had conferred important benefits on the British army; and it may be asserted, without fear of contradiction, that such palpable public neglect could not have occurred in the case of any individual in any other walk of life, having a moiety even of the intellectual and moral ascendency, or the claims of hard, honourable, and faithful services, possessed by ROBERT JACKSON." P. cxx.

Of "The Life" of the Author of the remarkable work before us, we shall only say, in conclusion, that it is altogether worthy its subject. No higher praise, we believe, could be bestowed on it. Of the work itself, we cannot, in this place, be expected to take a formal notice; but, if the declaration of Mr. Guthrie be true—(and who shall doubt it)—that "The Surgeon of a regiment learns the duty of a Soldier in addition to that of a Doctor, and a Military Surgeon ought to know the one just as well as the other;"—then, we say that, no medical officer in the public service should be unprovided with "the GREAT MILITARY WORK" of Robert Jackson.

